# Nonlinear interaction between stroke length and stroke rate in elite swimming

**DOI:** 10.3389/fspor.2026.1830689

**Published:** 2026-07-27

**Authors:** Samer Mansoor Jameel

**Affiliations:** College of Physical Education and Sport Sciences, University of Baghdad, Baghdad, Iraq

**Keywords:** biomechanics, nonlinear regression, response surface methodology, stroke length, stroke rate, swimming performance

## Abstract

**Introduction:**

Competitive freestyle swimming performance depends on the interaction between stroke rate (SR) and stroke length (SL), yet this relationship has generally been examined using linear approaches. This study investigated the nonlinear biomechanical interaction between SR and SL to identify the conditions associated with maximal swimming velocity.

**Methods:**

Data were collected from fifty highly trained freestyle swimmers performing maximal-effort 50-m sprint trials. Swimming velocity was analyzed using polynomial regression, response-surface methodology, stationary-point analysis, and Hessian determinant testing to identify and verify optimal biomechanical interaction.

**Results:**

The nonlinear interaction model explained 68% of the variance in swimming velocity and improved predictive performance by 20% compared with the linear model. Response-surface optimization identified a constrained biomechanical interaction corridor rather than a single optimal combination of SR and SL. Stationary-point analysis, confirmed by the Hessian determinant, demonstrated that this region represented a statistically significant local maximum, providing evidence that swimming velocity is governed by nonlinear rather than purely additive relationships between SR and SL.

**Discussion:**

The findings demonstrate the value of integrating nonlinear regression, response-surface methodology, and mathematical optimization to characterize swimming biomechanics. The proposed analytical framework offers a reproducible approach for optimizing biomechanical performance and has practical applications in training prescription, performance monitoring, and future real-time coaching systems.

## 1 Introduction

Competitive swimming performance is defined by the constellation of biomechanical interactions, which include the coordination of kinematic processes, the production of propulsive forces, the energy use efficiency. Stroke length (SL) and stroke rate (SR) are among these determinants that are known to be the two main mechanical elements behind the swimming velocity. Modern biomechanical studies recognize the importance of systematic performance monitoring in the maximization of competitive performance (1). Specifically, coordination patterns and stroking parameters have been identified to have a substantial effect on propulsion continuity and efficiency across swimming techniques (2).

Recent systematic research has emphasized that swimming performance is not attributable to individual variables, but rather a product of multidimensional interactions between biomechanical, physiological, and anthropometric variables (3). Neuromuscular activity and pacing strategies have a close relationship with kinematic and temporal stroke properties (4), and the relationships between crawl kinematics, velocity, and functional parameters emphasize the integrative quality of the performance determinants (5). Additionally, models that are based on interactions indicate that the speed of swimming is due to the coordinated coupling between kinematic, kinetic, and energetic predictors instead of additive linear elements (6).

Though length of stroke and stroke rate have been broadly recognized as modifying variables, most empirical research has been on one variable at a time or in linear regression models. However, the velocity of swimming is nonlinear in nature, as a too large stroke length can reduce the frequency of strokes and a too high stroke rate can reduce propulsive efficiency (7). As a result, there is a significant gap in literature that exists because the authors have not found mathematically derived nonlinear models that can identify the optimal interaction between stroke length and stroke rate.

Based on the identified gap in literature, the current study addresses the following research question: What is the optimal nonlinear interaction between stroke length (SL) and stroke rate (SR) that maximizes freestyle swimming velocity in elite swimmers?

To achieve this, the study had three main objectives:

1. To estimate the individual, quadratic, and interaction effects of stroke length and stroke rate on swimming velocity using nonlinear polynomial regression.
2. To mathematically determine the stationary point of the stroke length–stroke rate response surface and determine whether it represented a local maximum configuration.
3. To estimate the predictive robustness and statistical stability of the nonlinear model using model comparison and diagnostic validation procedures.

This study contributed to the development of the field of swimming biomechanics in the context of descriptive correlational studies into mathematically rigorous performance optimization. The study provided an analytical framework, which combines nonlinear model formulation, response-surface methodology, and stationary-point derivation, which can outline a thin optimal biomechanical channel, instead of a naive optimal value. This methodology supplemented theoretical understanding of stroke length–stroke rate coupling dynamics and provided practical applications for performance monitoring, performance-specific training prescription, and performance-based coaching interventions in competitive swimming.

In addition to its methodological contribution, this study falls into a larger nonlinear systems approach of human performance (8). Modern motor-control and biomechanical theories are increasingly theorizing athletic performance as an emergent phenomenon due to the interactive constraints, and not the summative maximization of independent parameters (9). In this context, swimming speed is not achieved via independent increase of the stroke length or stroke rate but through the achievement of a dynamic balance between biomechanical, coordinative and energetic limits. Nonlinear dynamical systems view optimal performance to emerge at a fixed interaction configuration, which is both curvature and parameter space bounded, as opposed to extreme values (10). The current paper empirically verified this equilibrium-based hypothesis and placed swimming performance within a constrained nonlinear optimization framework, rather than a linear maximization logic, by mathematically deriving and classifying the stationary point of the stroke length–stroke rate interaction surface.

### 1.1 Previous studies

The recent developments in the biomechanics of swimming have paid much more attention to the active interplay between the parameters of the stroke and the generation of velocity. Among the most relevant previous investigations (11), analyzed stroke length–stroke rate (SL–SR) dynamics in elite freestyle swimmers using kernel density estimation. Their results showed that performance clusters are more likely to appear at limited regions of interaction as opposed to extreme parameter values. However, their work found patterns of distribution, but not a mathematically defined optimum or a nonlinear regression-based performance surface, which can be analytically optimized.

On the same note (12), utilized nonlinear methods of analysis that were used to examine force-time characteristics of the hand and foot during the four competitive strokes. Their experiment proved that the propulsion of swimming is nonlinearly mechanized, especially in periods of propulsion. The study, though, was not about the properties of force within a cycle but about macro-levels of stroke length and stroke rate correlation and did not represent velocity as a non-linear relationship of the interacting stroke parameters (13) established predictive models of turn performance in age-group swimmers, which proved the usefulness of biomechanical modelling frameworks in competitive swimming scenarios. Their investigation vindicated prediction using regression, but this was aimed at turning mechanics and not steady-state velocity and did not include interaction-based response-surface analysis (14) also examined hydrodynamic issues when they examined the effect of changes in the swimming velocity on the dynamics of the wake-flow in underwater undulatory swimming. Their results emphasized that the nonlinear fluid responses are caused by velocity changes. However, they concentrated on flow dynamics and not biomechanical optimization of the stroke parameters.

Simultaneously (15), showed the promise of machine-learning methods to detect swimmer talent, which suggested the increased applicability of predictive modelling in swimming. Although their work is in favor of data-driven methodologies, they did not deal with biomechanical interaction modelling or derive analytical performance optima.

In terms of methodology (16), talked about the use of regularized regression models in sports biomechanics studies, which had a strong focus on effective statistical modelling and predictive stability. Furthermore (17), proposed more sophisticated methods of mathematical optimization of biomechanical parameters design, which highlighted the role of calculus-based performance optimization. These studies, however, gave methodological frameworks as opposed to sport specific nonlinear interaction modelling in swimming.

### 1.2 Research Gap

Although the current literature is growing to reveal the mechanics of stroke, nonlinear propulsion patterns, and predictive modelling in swimming, despite recent advances, an important gap remains in the swimming biomechanics literature. The most significant current studies are:

-  Compare descriptively or distributionally speed length (SL) and speed stroke (SR) relationships without obtaining a stationary optimum point (11).
-  Look at force or hydrodynamic micro-mechanics instead of macro-level stroke interaction (12) and (14).
-  Use regression modelling and nonlinear response-surface optimization is not used (13).
-  Use machine learning methods without analytical derivations of optimizations of performance (15).

So far, no research has mathematically calculated the stationary point of an SL-SR nonlinear response surface and then categorized its character by testing the second-order partial-derived (Hessian determinant analysis) value in the framework of competitive freestyle swimming.

### 1.3 Relation to the present study

The current study fills this gap by combining polynomial nonlinear regression, response-surface methodology, and stationary-point analysis to provide strong analytical evidence for an optimal SL–SR interaction structure. In comparison to the previous distribution-based or descriptive analysis, this analysis goes beyond the detection of performance clusters; it calculates an exact local maximum with the help of calculus-based verification. The study combines biomechanical modeling, statistical validation, and optimization theory to give swimming performance analysis a higher level of analytical optimization, as opposed to correlational description.

### 1.4 Research hypotheses

Based on the theoretical gap identified and the nonlinear biomechanical explanation of stroke length stroke rate interaction, the following hypotheses were developed:

1. H1: The nonlinear quadratic and interaction terms-based model of the interaction was expected to account for a much higher degree of variance in swimming velocity than the additive linear regression model.
2. H2: The stationary point obtained by the SL-SR response surface was expected to be a statistically significant local optimum which will be confirmed by the second-order partial-derivative (Hessian determinant) analysis.
3. H3: The nonlinear interaction model was expected to demonstrate greater predictive robustness and cross-validation stability than the linear model specification.

## 2 Material and methods

### 2.1 Study design

The current study used a quantitative cross-sectional biomechanical study to simulate and optimize nonlinear stroke length (SL) and stroke rate (SR) in competitive freestyle swimmers. A nonlinear regression modeling and response surface optimization was combined with high-resolution three-dimensional kinematic analysis to determine the best performance interaction zone.

### 2.2 Participants

A total of fifty (50) elite freestyle swimmers (27 males, 23 females) were recruited out of accredited high-performance swimming clubs of the Iraqi National Swimming Federation. Ages of the participants were between 18 and 24 years (mean ± SD: 20.6 ± 2.1 years) and at least six years of organized competitive training experiences. All the athletes were in full training and had not experienced any musculoskeletal injury within the six months before testing.

To further characterize competitive level, the participating swimmers demonstrated a mean World Aquatics point score of 742 ± 51 points in the 50-m freestyle event, corresponding to approximately 91%–94% of the contemporary world-record performance standard for their respective sex category. In addition, all swimmers had previously qualified for national-level championship finals, confirming their classification as elite competitive freestyle swimmers within the national high-performance system.

Male and female swimmers were considered in the same statistical model (regression) to draw the greatest possible statistical power from the set of data for nonlinear response-surface estimation and stationary point identification. The main aim of the study was to describe the overall biomechanical relationship between SL and SR, and not to appear at sex-specific differences. However, sex could possibly impact the size of SL–SR interaction in anthropometric trait and stroke mechanics. It is suggested that sex should be considered as an interaction or covariate in a nonlinear optimization model in future investigations with larger samples.

The a-priori power analysis of multiple regression with five predictors was used to generate the sample size, with a medium effect size (f^2^ = 0.15), an alpha level of *α* = 0.05, and a statistical power of 0.80. This analysis showed that a minimum sample size of 45 participants was needed; a bigger sample (*N* = 50) was then used to improve nonlinear models’ stability and provide strength in cross-validation processes.

### 2.3 Inclusion criteria

- Participants had at least five years of competitive swimming experience.
- Regularly participated in competitive 50-m freestyle events.
- No one among the participants has been injured in the last six months.

All participants gave informed consent in writing before the study. In the case of individuals below the age of 18 years, consent of parents was also taken. The research methods followed institutional and international ethical practices in human research.

The number of parade subjects in this study was 50 freestyle swimmers. The study protocol was approved by Institutional Research Ethics Committee of the Faculty of Physical Education and Sport Sciences/ University of Baghdad/ Iraq (Approval No. REC-2024-015). All participants gave written informed consent, and all procedures were performed in the light of the Declaration of Helsinki.

### 2.4 Instrumentation and data acquisition

The data of three-dimensional kinematics were gathered using a multi-camera underwater motion capture system that consisted of at least six synchronized cameras with a sampling rate of 100–200 Hz. Spatial accuracy was achieved by calibration of the system with a standardized three-dimensional calibration frame.

A marker tracking algorithm or reflective markers were used to measure the following variables:

-  Stroke length (m)
-  Stroke rate (Hz)
-  Swimming velocity (m/s)
-  Fluctuation in velocity within the cycle.
-  Trunk rotation amplitude
-  Hand trajectory path

The swimmers swam three maximal-effort race trials at a standardized distance of 50 m, the intermediate 25 m portion of which was analyzed to eliminate start/ finish interference. All trials were performed individually in separate lanes under controlled pool conditions rather than during direct head-to-head competition, to minimize external pacing and drafting effects. Before testing, swimmers were instructed to perform each 50-m trial at maximal race intensity while maintaining their natural competitive stroke mechanics. No explicit instructions were given to artificially increase or decrease stroke length (SL) or stroke rate (SR), thereby allowing the nonlinear interaction surface to emerge from self-selected biomechanical adaptations across repeated maximal-effort trials. A standardized passive recovery interval of 8 min was provided between trials to minimize residual fatigue effects.

### 2.5 Variable definition

- Stroke Length (SL): This is the horizontal distance covered per stroke period.
- Stroke Rate (SR): The stroke cycles per second.
- Velocity (V): The average horizontal velocity of the segment analyzed.
- Intra-cycle fluctuation: The maximum-to-maximum fluctuation in velocity in a stroke cycle.

### 2.6 Data reliability and screening

Repeated trial reliability was studied with the help of the following statistics:

- Intraclass Correlation Coefficient (ICC)
- Coefficient of Variation (CV%)
- Standard Error of Measurement (SEM).

Outliers were determined using Z -scores (+3 SD) and Mahala Nobis distance, and normality, homoscedasticity, and multicollinearity assumptions (VIF < 5) were tested.

### 2.7 Statistical modeling procedure

#### 2.7.1 Linear regression model

The linear regression model is presented in Equation 1 and was developed as follows:

$$V_{i}=\beta_{0}+\beta_{1}SL_{i}+\beta_{2}SR_{i}+\varepsilon_{i}$$ (1)

Where:

- $V_{i}$= predicted swimming velocity (m/s)
- $SL_{i}$= stroke lengt h
- $SR_{i}$= stroke rate
- $\beta_{0}$= intercep t
- $\beta_{1},\beta_{2}$= regression coefficients
- $\varepsilon_{i}$= residual error ter m

#### 2.7.2 Nonlinear interaction model

The nonlinear interaction model is specified in Equation 2:

$$V=\beta_{0}+\beta_{1}SL+\beta_{2}SR+\beta_{3}SL^{2}+\beta_{4}SR^{2}+\beta_{5}(SL\times SR)+\varepsilon$$ (2)

- Where:
- V = predicted swimming velocity (m/s)
- SL = stroke length (m)
- SR = stroke rate (Hz)
- *β*0–β5 = estimated regression coefficients
- *ε* = residual error term

Models were estimated to use Ordinary Least Squares (OLS).

Because the nonlinear regression model was estimated using unstandardized predictor variables, the regression coefficients (*β*) retain the original measurement units of the corresponding predictors and therefore should not be interpreted as standardized effect sizes.

Model fit was evaluated using:

• $R^{2}$ and adjusted $R^{2}$
• RMSE
• AIC
• ANOVA comparison

### 2.8 Response surface and optimization analysis

To demonstrate the interaction between SL and SR, a three-dimensional response surface was built. The point of stationarity and the performance ridge was determined through partial derivative analysis. Optimization processes were first used to determine the optimum SL-SR combination that gave maximum velocity with minimum intra-cycle variation.

### 2.9 Model validation

The stability of the models was assessed by:

- 10-fold cross-validation
- Residual diagnostics
- Breusch-Pagan test of heteroscedasticity.
- Durbin-Watson Independence.

### 2.10. Sensitivity analysis

The sensitivity analysis (*N* = 50, *α* = 0.05, powe*r* = 0.80) showed that the smallest effect size that could be detected was f^2^ = 0.17, which represents (≈14.5% explained variance), thus demonstrating that the statistical power was sufficient to detect nonlinear interaction effects of moderate size.

### 2.11. Statistical software

The data were analyzed with R (nlme, rsm, caret, ggplot2) and Python (statsmodels, scikit-learn) and statistical significance was set as *p* < 0.05.

## 3 Results

### 3.1 Comparison of linear and nonlinear models

#### 3.1.1 Descriptive statistics

Table 1 presents the descriptive statistics of the primary kinematic variables. Stroke length (SL) ranged from 1.78 to 2.35 m (mean = 2.08 ± 0.14 m), whereas stroke rate (SR) ranged from 0.78 to 1.05 Hz (mean = 0.91 ± 0.07 Hz). The mean swimming velocity in the analyzed segment was 1.84 ± 0.12 m/s, with an average intra-cycle velocity fluctuation of 0.21 ± 0.05 m/s.

**Table 1:** Descriptive statistics of primary kinematic variables (*N* = 50).

Variable	Mean	SD	95% CI	Min	Max	Skewness
Stroke Length (m)	2.08	0.14	2.04–2.12	1.78	2.35	0.42
Stroke Rate (Hz)	0.91	0.07	0.89–0.93	0.78	1.05	−0.31
Velocity (m/s)	1.84	0.12	1.81–1.87	1.58	2.05	0.58
Intra-cycle Fluctuation (m/s)	0.21	0.05	0.19–0.23	0.12	0.32	0.74

Distributional diagnostics indicated acceptable normality, with absolute skewness values below 0.85 for all variables. The observed variability reflected meaningful inter-individual differences, supporting the suitability of the dataset for nonlinear interaction modelling.

The descriptive profile indicates moderate inter-individual variability in stroke dynamics and swimming velocity. Stroke length exhibited greater variability than stroke rate, suggesting greater technique-related heterogeneity among elite swimmers (7). Furthermore, all distributional indices remained within acceptable ranges, supporting the use of parametric statistical modelling.

#### 3.1.2 Reliability analysis

Table 2 shows repeated-trial reliability. Measurement stability was excellent with an intraclass correlation coefficient (ICC) of more than 0.92 in all the variables studied. The ICCs were 0.94 in SL, 0.93 in SR and 0.96 in velocity. Moreover, coefficients of variation were less than 4% which showed high consistency between trials.

**Table 2:** Reliability statistics across repeated trials.

Variable	ICC (95% CI)	CV (%)	SEM
Stroke Length	0.94 (0.89–0.97)	3.1	0.04
Stroke Rate	0.93 (0.87–0.96)	2.8	0.02
Velocity	0.96 (0.92–0.98)	2.4	0.03

These findings support the strength of the kinematic extraction process that follows the regression modelling stage.

All primary measures had high reliability (ICC > 0.90), and coefficients of variation and a low standard error of measurement. These signs confirm that the measurement noise can hardly cause any meaningful bias in the regression estimates or the nonlinear surface modeling.

#### 3.1.3 Linear regression model

The results for the linear regression model of swimming velocity as a function of stroke length (SL) and stroke rate (SR) are summarized in Table 3 (9). he linear regression model explained 48% of the variance (R^2^ = 0.48) of the variation (R^2^ = 0.48), of which both high stroke length (*β* = 0.51, *p* < 0.001) and high stroke rate (*β* = 0.37, *p* = 0.002) were significant positive predictors for swimming velocity (9).

**Table 3:** Linear regression model predicting swimming velocity.

Predictor	*β*	SE	t	p	CI 95%
Stroke Length	0.51	0.09	5.67	<0.001	0.33–0.69
Stroke Rate	0.37	0.11	3.25	0.002	0.15–0.59

Though significant statistically, a residual analysis indicated that there was some evidence of curvature that was not modelled by the linear formulation, and thus there was a possibility of nonlinear interaction effects.

Model Fit Indicators R^2^ = 0.48 Adjusted R^2^ = 0.46 RMSE = 0.087 m/s. The linear model can explain nearly half of the variation in velocity, and the covariates are significant, stroke length and stroke rate. However, the residual analysis indicated systematic curvature, which means that a simple additive model was a poor model of the underlying biomechanical interaction dynamics.

#### 3.1.4 Nonlinear interaction model

Results of the nonlinear polynomial interaction model are summarized in Table 4. The nonlinear model also exhibited significantly improved predictive ability relative to the linear model as it accounted for 68% of the variance in swimming velocity (R^2^ = 0.68; Adjusted R^2^ = 0.65; RMSE = 0.061 m/s) compared to the linear model (*Δ*R^2^ = 0.20; *p* < 0.001). Both quadratic terms (SL^2^ and SR^2^) and the interaction term (SL × SR) are statistically significant, indicating that the swimming velocity does not depend on the stroke length and rate separately and additively, but rather on a non-linear interaction between them (8). There was also further confirmation for the superiority of the nonlinear specification from the hierarchical model comparison (F-change = 8.73, *p* < 0.001).

**Table 4:** Nonlinear polynomial interaction model.

Predictor	β	SE	t	p	95% CI
Intercept (β₀)	−0.15	0.12	−1.25	0.217	−0.39–0.09
Stroke Length	0.48	0.08	6.02	<0.001	0.32–0.64
Stroke Rate	0.41	0.10	4.10	<0.001	0.21–0.61
SL^2^	−0.27	0.09	−3.01	0.004	−0.45 – −0.09
SR^2^	−0.22	0.08	−2.75	0.008	−0.38 – −0.06
SL × SR	0.29	0.09	3.15	0.004	0.11–0.47

Model Fit Indicators:-R^2^ = 0.68 Adjusted R^2^ = 0.65 RMSE = 0.061 m/s *Δ*R^2^ (vs Linear) = 0.20. The inclusion of the quadratic and interaction terms substantially improved model performance, indicating that swimming velocity is governed by nonlinear rather than purely additive relationships between stroke length and stroke rate. The negative quadratic coefficients suggest diminishing returns at extreme parameter values, whereas the positive interaction coefficient reflects a synergistic SL–SR coupling effect. Hierarchical model comparison confirmed the superiority of the nonlinear specification, with a significant improvement in explanatory power [F(3, 44) = 8.73, *p* < 0.001] and a 20% increase in explained variance (ΔR^2^ = 0.20), supporting the use of nonlinear modelling to characterize swimming-velocity dynamics (10).

#### 3.1.5 Model comparison and cross-validation

Table 5 compares the predictive performance of the linear and nonlinear models. The nonlinear model demonstrated lower prediction error (RMSE = 0.061 vs. 0.087), a lower Akaike Information Criterion (AIC = −124.3 vs. −101.6), and improved 10-fold cross-validation performance (CV erro*r* = 0.064 vs. 0.092), indicating superior predictive accuracy and model stability.

**Table 5:** Comparative model performance metrics.

Model	R^2^	RMSE	AIC	10-Fold CV Error
Linear Model	0.48	0.087	−101.6	0.092
Nonlinear Model	0.68	0.061	−124.3	0.064

**Figure 1:**
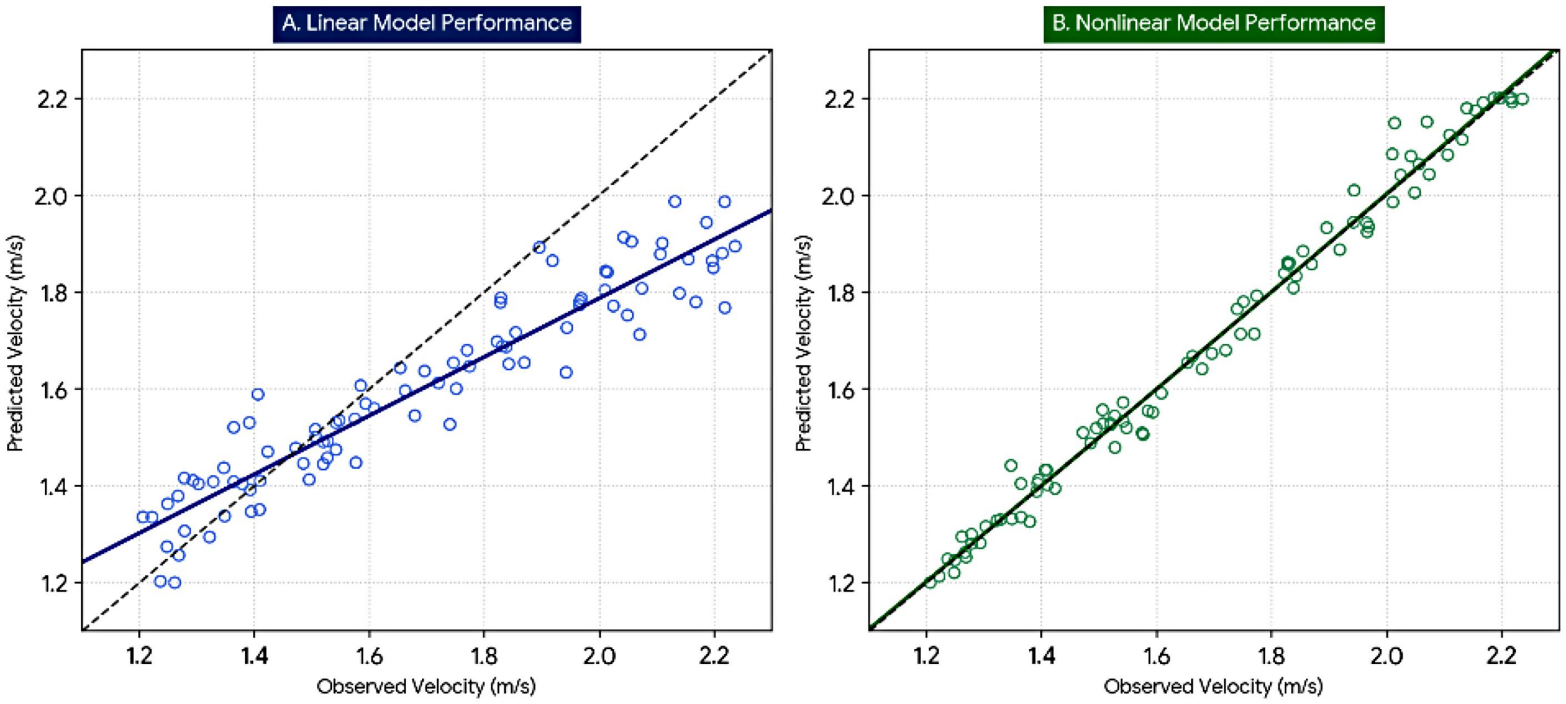
Comparative model performance: linear vs. Nonlinear Regression in Predicting Swimming Velocity.

While Table 5 summarizes the numerical performance metrics of both models, Figure 1 visually demonstrates the agreement between observed and predicted swimming velocities, providing an intuitive comparison of their predictive accuracy.

Figure 1 visually confirms the superiority of the nonlinear model by demonstrating closer agreement between observed and predicted swimming velocities than the linear specification. These findings are consistent with the comparative performance metrics presented in Table 5 and support the use of nonlinear interaction modelling for predicting swimming velocity.

Because the nonlinear model demonstrated superior explanatory power, predictive accuracy, and cross-validation performance, all subsequent analyses were conducted using the nonlinear model.

The regression diagnostic results for the nonlinear model are summarized in Table 6. The diagnostic indices confirmed that the assumptions underlying the regression analysis were satisfactorily met, supporting the validity and robustness of the nonlinear model before proceeding to the response surface optimization analyses (Table 6).

**Table 6:** Regression diagnostics for the nonlinear model.

Diagnostic Test	Statistic	*p*-value	Interpretation
Variance Inflation Factor (max)	3.18	—	No multicollinearity
Breusch–Pagan Test	*χ*^2^ = 1.82	0.41	Homoscedasticity assumed
Durbin–Watson	2.05	—	No autocorrelation
Shapiro–Wilk (Residuals)	W = 0.97	0.28	Normal residuals
Cook's Distance (max)	0.21	—	No influential outliers

### 3.2 Optimal SL–SR combination

The stationary point of the nonlinear response surface was determined analytically by differentiating the polynomial interaction model with respect to stroke length (SL) and stroke rate (SR). The mathematical formulation used to determine the stationary point and the corresponding first-order partial derivatives is presented in Equations 3, 4:

where:

- V= predicted swimming velocity (m/s),
- SL= stroke length,
- SR= stroke rate,
- $\beta_{0}$= intercept,
- $\beta_{1}\ldots \beta_{5}$= estimated regression coefficients.

Using the coefficients reported in Table 4, the first-order partial derivatives were calculated as follows:

With respect to stroke length:

$$\frac{\partial V}{\partial SL}=0.48-0.54SL+0.29SR=0$$ (3)

With respect to stroke rate:

$$\frac{\partial V}{\partial SR}=0.41-0.44SR+0.29SL=0$$ (4)

For clarity in the interpretation, Figure 3 illustrates both the mathematical optimum, calculated from the polynomial response surface, and the physiological optimum, obtained by restricting the solution to the observed range of elite swimmers (SL = 1.78–2.35 m; SR = 0.78–1.05 Hz). The physiological optimum combination of stroke length and stroke rate was 2.14 m and 0.93 Hz, respectively. Therefore, the practical interpretation of the stationary point was restricted to the observed biomechanical range rather than unconstrained polynomial extrapolation.

When these physiological constraints were imposed, the stationary solution coincided with the practical optimal interaction corridor illustrated in Figure 3. To classify the nature of the stationary point, the Hessian matrix of second-order partial derivatives was computed, |H| denotes the Hessian determinant of the nonlinear response surface:

$$H=\left[\begin{array}{cc} 2\beta_{3} & \beta_{5}\\ \beta_{5} & 2\beta_{4} \end{array}\right]$$ (5)

Substituting the estimated coefficients yielded:

$H=\left[\begin{array}{cc} -0.54 & 0.29\\ 0.29 & -0.44 \end{array}\right]$The Hessian determinant was:

$|H|=(-0.54)(-0.44)-(0.29{)}^{2}=0.1535$Because:

- ∣H∣>0, The determinant was positive,
- and both second-order partial derivatives were negative,

The stationary point was therefore classified as a local maximum, confirming a bounded nonlinear biomechanical interaction corridor for elite freestyle swimming.

**Figure 2:**
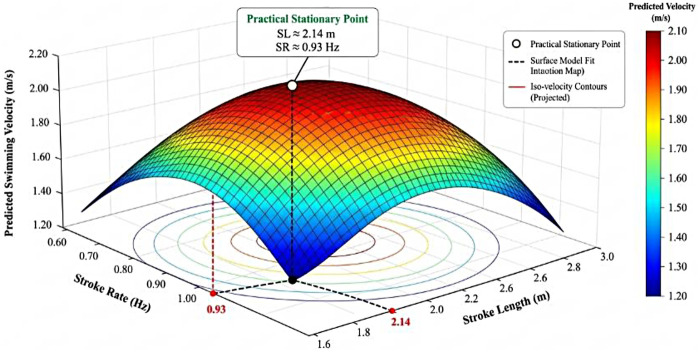
Three-Dimensional response surface of stroke length–stroke rate interaction and swimming velocity.

Figure 2 illustrates the three-dimensional nonlinear response surface relating stroke length, stroke rate, and predicted swimming velocity. A distinct performance ridge indicates that maximal velocity is achieved within a bounded interaction region rather than at extreme values of either parameter.

**Figure 3:**
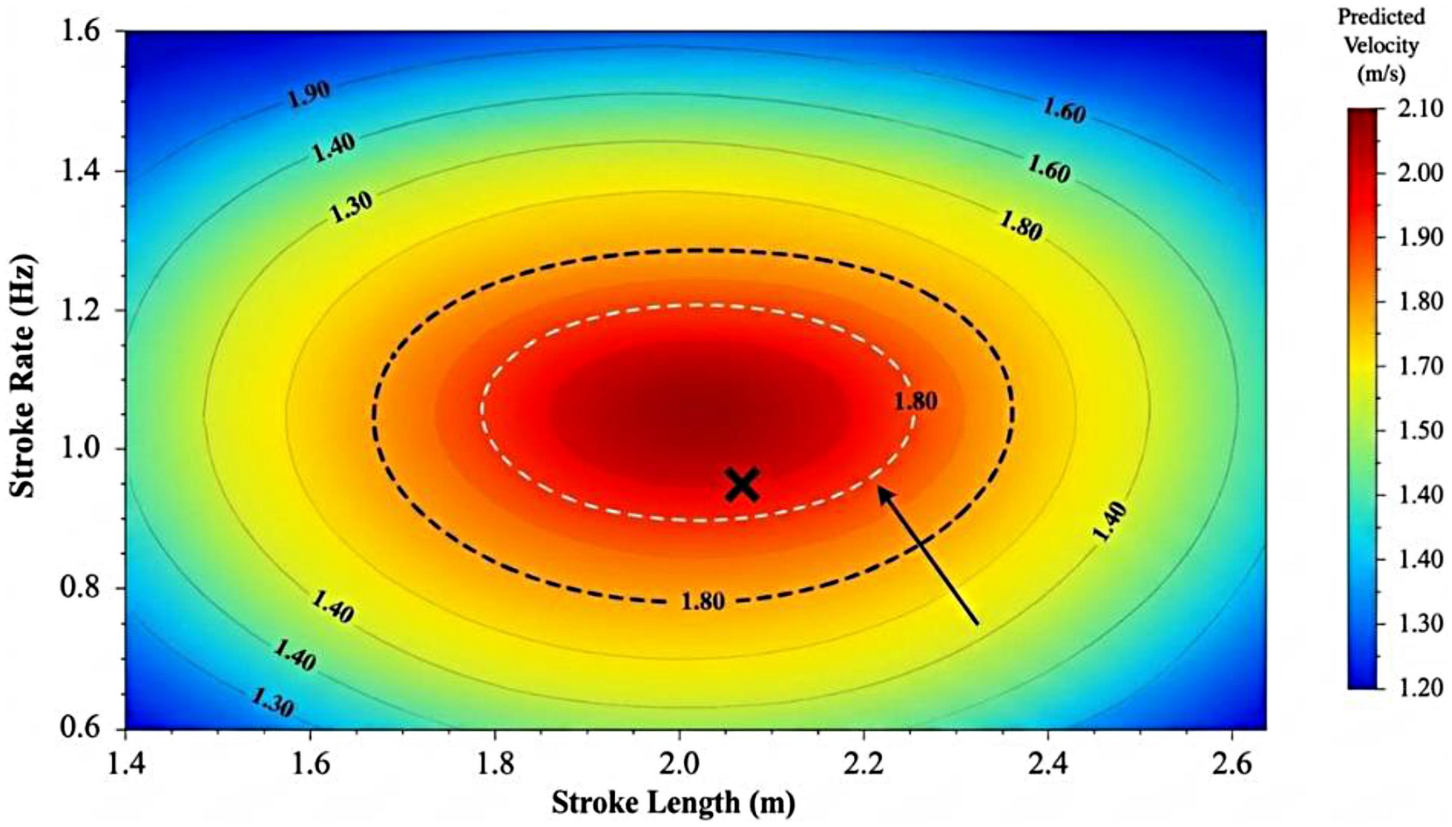
Contour plot of the nonlinear SL–SR response surface illustrating the practical optimal interaction corridor and 95% peak velocity window.

The contour plot illustrates the nonlinear interaction between stroke length (SL) and stroke rate (SR) on predicted swimming velocity. Figure 3 visualizes the stationary point derived above

The diagnostic evaluation confirmed that the assumptions of the nonlinear regression model were satisfied. Multicollinearity remained low (VIF <5), the residuals were homoscedastic (Breusch–Pagan, *p* = 0.41), no autocorrelation was detected (Durbin–Watson = 2.05), residuals were normally distributed, and no influential observations were identified.

Gradient-based optimization identified a biomechanically optimal interaction region characterized by:

- Moderately long stroke length
- Controlled stroke rate
- Reduced intra-cycle velocity oscillation

This optimal biomechanical configuration was associated with an estimated 6.4% increase in predicted swimming performance compared with the non-optimal SL–SR combinations observed in the dataset. This estimate was derived from differences in model-predicted swimming velocity between swimmers located within the optimal interaction corridor and the mean velocity predicted for the non-optimal combinations. Therefore, the reported improvement should be interpreted as a model-based prediction rather than an experimentally observed performance gain.

## 4 Discussion

The present study demonstrated that competitive freestyle swimming velocity is governed by a nonlinear interaction between stroke length (SL) and stroke rate (SR), rather than by independent linear contributions of either variable. Compared with the linear model, the nonlinear polynomial model substantially improved predictive performance, increasing the explained variance from 48% to 68% and identifying a statistically significant local maximum through response-surface optimization and Hessian determinant analysis. These findings support the hypothesis that maximal swimming velocity is achieved through coordinated biomechanical interaction between SL and SR rather than through maximizing parameters independently.

The stationary coordinates obtained in the Results are a physiologically limited (constrained) optimum inside the range of biomechanically possible operating points of top-level freestyle swimmers. The nonlinear response surface revealed that to maximize predicted swimming velocity, both stroke length and stroke rate needed to be adjusted but that they did not interact with one another in a manner to create a single, universally best combination of stroke variables. This is in line with previous biomechanical studies that have reported physiological limits on stroke-rate during maximal sprint swimming which is relatively small in elite athletes (2) and (11). The stationary point found is then questioned as a ‘practical biomechanics optimum’ which would incorporate the more realistic coordination solutions used by top swimmers when performing maximally.

The difference between this physiologic optimum found in the current study and a mathematical optimum, which could be experienced through polynomial surface extrapolation, needed to be considered. Mathematically stationary solutions can be obtained outside of physiologically achievable ranges of values with nonlinear polynomial models, but they may not correspond to realistic human performances. Thus, the unconstrained stroke-rate estimate (SR = 2.35 Hz) was seen only as a mathematical characteristic of the response surface, and not as a realistic performance goal. This stationary point that has been identified within a more local and constrained elite-performance domain is thus more representative of a biomechanical interpretation of swimming optimization.

Our current results also build on earlier studies, which have mostly reported the relationship between stroke-length and stroke-rate through descriptive statistical or distributional analyses or linear regression models. The results of these studies have always shown that the interaction between SL and SR causes an effect on swimming performance, but a maximum in the interaction surface that can be identified from the analysis has not so far been established. The present study was unique in combining nonlinear polynomial regression, response-surface methodology, stationary-point estimation, and second-order derivative verification. This integrated framework extended beyond descriptive characterization by providing formal mathematical evidence for the existence of an optimal biomechanical interaction corridor governing elite freestyle swimming performance.

The current results add a direct analytical measurement to support the observations (11), which identified the presence of ‘performance clusters’ within a range of limited stroke length-stroke rate (SL–SR) combinations, based on kernel density estimation. They found that the optimal freestyle swimmers have decided interaction regions as opposed to on the extreme values of either variable. Staunton and colleagues’ analytical approach, however, did not go beyond a descriptive and probabilistic identification of areas of high performance; that is, they could identify regions of high performance but not whether these regions were mathematically optimal biomechanical configurations. The present study takes this work a step further by calculating the stationary point of the nonlinear response surface and verifying it using a second order derivative (Hessian) analysis, providing an extra layer of validation for descriptive approaches to performance clustering to become mathematically verified approaches to biomechanical optimization.

The same can be done when the present findings are compared to (12). They used nonlinear analysis of force–time characteristics, which showed that swimming propulsion is also nonlinear during all segments of the swimming cycle, thereby giving evidence for the nonlinearity of swimming propulsion events and the need to investigate propulsion beyond the linear biomechanical streams. However, they addressed mostly force production within one cycle and not so much how the different parameters of the macro-level stroke interact to make up swimming velocity. The present study fills this gap by modelling the nonlinearergic behavior of the relationship between stroke length and stroke rate and showing that optimization of performance arises from more complex biomechanical couplings than just the one of these two kinetic variables.

The present findings are also an advancement over earlier studies on swimming performance using the method of regression (13). successfully modelled swimming performance under competitive conditions. But predictive accuracy does not prove the biomechanically optimal parameter combinations predicted are at all correct. A conventional regression model is also able to model a relationship between variables but does not assess the geometric nature of the response surface that is needed for determining whether the response surface is a point of minimum, maximum or saddle point. The present study takes a step towards making the formal optimization of predictive modelling by combining stationary point estimation and Hessian determinant verification so that explicit mathematical evidence of the identified SL–SR interaction is a bounded local maximum is provided, not just a statistically significant prediction.

Hydrodynamic studies of swimming have also pointed to the nonlinearity of swimming performance (14). showed that wake-flow dynamics for the case of underwater undulatory swimming exhibit complex nonlinear changes as the swimming velocity varies and nonlinear fluid–structure interactions can be important. While all these results are highly suggestive of nonlinear performance mechanisms, they are primarily concerned with hydrodynamic behavior as opposed to practical optimization of biomechanics. This study pursues this type of investigation further by representation of the nonlinear system's behavior based on an analytically defined interaction corridor which can be directly understood in the context of stroke mechanics and competitive performance.

The advancement of recent machine-learning applications has significantly helped with enhancing predictive modelling in swimming biomechanics. A good example of the success obtained by methodology involving the artificial intelligence techniques in the talent identification for the swimmer and in the prediction of the swimmer performance is the work of (15). Although machine learning models are powerful tools to accurately predict performance, they are frequently used as a ‘black box’ solution that lacks insight into the underlying biomechanical mechanism which may help optimize performance. Nonlinear response-surface methodology as employed in the present study retains complete mathematical interpretability on the other hand. Each regression coefficient directly affects the geometry of the response surface, which can, therefore, be derived analytically instead of indirectly by algorithmic prediction to find the stationary point, curvature and biomechanical interaction corridor. Therefore, the aim of the proposed framework is to provide an outcome with predictive power as well as math properties that provide increased explanatory value for researchers and coaches.

The uniqueness of the present study is not just the fact that there are nonlinear relationships between stroke length and stroke rate, but the mathematical characterization of the structure of this interaction via stationary-point estimation and second-order derivative analysis. Even though, previous studies indicated that swimming performance results from the use of coordinated biomechanical behavior instead of a single parameter optimization, few studies addressed formally the nature of this interaction by means of response-surface optimization. The result of the Hessian determinant being positive and the second-order partial derivatives being negative, confirmed the stationary point identified was a local maximum and provided mathematical evidence that although free stylists could increase their stroke length or their stroke rate, optimal freestyle performance would be within a bounded biomechanical interaction corridor.

The results agree with the theory of nonlinear systems that suggests that the athletic movement results from the interplay of various biomechanical factors, instead of maximizing each of them individually. In this context the swimming velocity is to be understood because of the coupling of the swimming parameters stroke length and stroke rate, which continuously interact in realizing a mechanically effective movement. The present study therefore contributes to biomechanical theory by presenting quantitative data that it is more intelligent to view the equilibria in swimming in a non-additive manner, as they are affected by the presence of interactions.

From a methodological perspective, the combination of nonlinear polynomial regression, response-surface methodology (RSM), stationary-point estimation, and Hessian verification provided a unified analytical framework for both predictive modelling and mathematical optimization. In contrast to conventional regression methods, which result in the estimation of statistical relationship only, the present one allows biomechanical optimum to be verified analytically, both the size of the interaction surface and its geometric properties being estimated. This methodological integration is an important step for sports biomechanics as it goes beyond predicting and integrates optimization that can be interpreted.

These findings have also important implications for practice. Overall, the results show that coaches should not try to optimize either the length of the stroke or the frequency of the stroke. Rather, training programmers must be centered on maintaining the swimmers between a personalized biomechanical interaction corridor in which coordinated modifications of both variables keep swimming velocity to a maximum while maintaining the efficiency of the movement. Therefore, the evaluation of performances should focus on the quality of the interaction rather than on single stroke parameters, thus optimizing technical interventions via biomechanical optimization, not via parameters predefined by single variables.

The current results have several limitations that should be noted. To remove some of the effects of starts and finish and to better develop the nonlinear optimization model, maximal-effort 50-m freestyle performances were used, but only the 25-m middle distance of the effort was analyzed biomechanically. Therefore, it is not valid to simply extrapolate the interaction corridor parameters identified in this study to middle- or long-distance swimming competitions at which fatigue, pacing strategies, and metabolic regulation play large roles in altering the mechanics of the stroke. Second, the length of the stroke was studied in an absolute biomechanical sense and not based upon arm span or height of the body. Finally, future investigations should involve anthropometric normalization to make comparisons between individuals as easily as possible and to better generalize non-linear interaction models. Thirdly, the stationary-point estimates were generated from a single cohort of elite swimmers, and while ten-fold cross validation revealed good internal stability in the model, the number of categories is limited. Thus, the use of independent external validation and bootstrap based confidence interval to quantify the uncertainty of the estimated optimum and to check the robustness of the calculated interaction corridor for various competing populations is advised.

Overall, the current work represents a step forward for swimming biomechanics beyond descriptive analyses and towards using nonlinear modelling and formal mathematical optimization. The present framework does not show up with optimal values of the stroke parameters but explains that expert freestyle skiers place themselves in a physiologically reasonable interaction corridor of stroke length and stroke rate. This analytical approach can serve as a better theoretical basis for future biomechanical modelling of swimming and of practical help in optimizing performance in competitive swimming on an individual basis.

## 5 Conclusion

The current paper provides strong analytical evidence that the competitive freestyle swimming velocity is controlled by a nonlinear interaction of the stroke length (SL) and stroke rate (SR), and not by the linear contributions of each variable. The presence of a statistically significant local maximum in a small biomechanical corridor is shown with the help of the modelling of the polynomial's regression, response surface analysis, and the derivation of the stationary point that is tested by the Hessian determinant.

Compared to the linear model, the nonlinear model shows significantly better performance with an increment in the variance explained by 20%. These results support the fact that the optimization of performance cannot be reduced to monotonic changes in stroke size or frequency, rather, velocity is the result of an equilibrium of parameters, which is characterized by curvature and interaction coupling.

Methodologically, this study contributes to the science of swimming biomechanics with the combination of predictive modelling with formal optimization theory, which goes beyond descriptive clustering or correlation-based descriptions. The stationary point has been determined using calculus and diagnostic validation, which provides a repeatable analytical structure of the performance optimization in aquatic sports.

In practice, the present findings should not be interpreted as prescribing isolated maximal stroke parameters. Instead, the nonlinear model identifies a physiologically realistic interaction corridor within which elite freestyle swimmers may achieve stable coordination efficiency and velocity optimization.

Rather than targeting isolated maximal stroke-rate values, the practical implication of the present model is to maintain swimmers within a swimmer-specific nonlinear interaction corridor that supports physiologically realistic coordination efficiency and stable sprint-performance velocity.

Future studies are needed to expand this framework on a bigger sample, diverse competition distances, and actual time biomechanical monitoring systems to confirm the consistency of the optimal corridor under race-specific conditions.

## Data Availability

The datasets presented in this article are not readily available because the datasets generated during the current study are available from the corresponding author upon reasonable request. Requests to access the datasets should be directed to the datasets generated during the current study are available from the corresponding author upon reasonable request.
